# Abnormalities in structural covariance of cortical gyrification in schizophrenia

**DOI:** 10.1007/s00429-014-0772-2

**Published:** 2014-04-26

**Authors:** Lena Palaniyappan, Bert Park, Vijender Balain, Raj Dangi, Peter Liddle

**Affiliations:** 1 Division of Psychiatry and Applied Psychology, University of Nottingham, Room-09, C Floor, Institute of Mental Health Building, Triumph Road, Nottingham, NG7 2TU England, UK; 2 Centre for Translational Neuroimaging in Mental Health, Institute of Mental Health, Nottingham, UK; 3 Early Intervention in Psychosis, Nottinghamshire Healthcare NHS Trust, Nottingham, UK; 4 Penticton Regional Hospital, 550 Carmi Avenue, Penticton, V2A 3GS UK; 5 Lancashirecare NHS Foundation Trust, Preston Road, Chorley, PR7 1PP UK

**Keywords:** Gyrification, Segregation, Integration, Connectome, Centrality, Topology and graph theory

## Abstract

The highly convoluted shape of the adult human brain results from several well-coordinated maturational events that start from embryonic development and extend through the adult life span. Disturbances in these maturational events can result in various neurological and psychiatric disorders, resulting in abnormal patterns of morphological relationship among cortical structures (structural covariance). Structural covariance can be studied using graph theory-based approaches that evaluate topological properties of brain networks. Covariance-based graph metrics allow cross-sectional study of coordinated maturational relationship among brain regions. Disrupted gyrification of focal brain regions is a consistent feature of schizophrenia. However, it is unclear if these localized disturbances result from a failure of coordinated development of brain regions in schizophrenia. We studied the structural covariance of gyrification in a sample of 41 patients with schizophrenia and 40 healthy controls by constructing gyrification-based networks using a 3-dimensional index. We found that several key regions including anterior insula and dorsolateral prefrontal cortex show increased segregation in schizophrenia, alongside reduced segregation in somato-sensory and occipital regions. Patients also showed a lack of prominence of the distributed covariance (hubness) of cingulate cortex. The abnormal segregated folding pattern in the right peri-sylvian regions (insula and fronto-temporal cortex) was associated with greater severity of illness. The study of structural covariance in cortical folding supports the presence of subtle deviation in the coordinated development of cortical convolutions in schizophrenia. The heterogeneity in the severity of schizophrenia could be explained in part by aberrant trajectories of neurodevelopment.

**Electronic supplementary material:**

The online version of this article (doi:10.1007/s00429-014-0772-2) contains supplementary material, which is available to authorized users.

## Introduction

A substantial body of evidence supports the hypothesis that schizophrenia is a developmental disorder in which the cerebral connectivity and morphology are disturbed (Rapoport et al. 2012). Investigation of the cortical morphology is potentially informative about pathological deviations in neurodevelopment (Gay et al. 2012). In particular, neuroimaging and post-mortem studies report abnormal cortical folding in schizophrenia (White et al. 2003; Harris 2004; Wheeler and Harper 2007; Bonnici et al. 2007; Cachia et al. 2008; Schultz et al. 2010, 2011). Whole brain vertex wise localization studies note reduced gyrification in several brain regions including insula, parieto-temporal region, precuneus, lateral prefrontal cortex and precentral region, and increased gyrification in anterior aspect of prefrontal cortex (Nesvåg et al. 2014; Palaniyappan and Liddle 2012). Further, the longitudinal trajectory of regional gyrification deviates from that of age-matched peers without schizophrenia (Palaniyappan et al. 2013a). This suggests that the cross-sectional observations of altered regional gyrification in schizophrenia can be linked to maturational disturbances (White and Hilgetag 2011).

While comparing diagnostic groups with mass univariate whole brain analysis reveal localized regional changes in a ‘lesional’ sense, this approach fails to quantify the relationship between concomitant changes in different brain areas. Crucial information about abnormalities in the integrated development of the brain as a connected system can be gathered by studying the covariance of morphology. Graph-based approaches provide a powerful mode of finding subtle differences in brain organization (Bullmore and Sporns 2009). In particular, morphological networks based on anatomical covariance among brain regions capture an important aspect of developmental maturation crucial for understanding the pathophysiology of psychotic disorders (Alexander-Bloch et al. 2013a; Evans 2013). Direct evidence linking anatomical covariance to coordinated brain development is beginning to emerge in recent times (Raznahan et al. 2011; Alexander-Bloch et al. 2013a, b). Graph theory offers a powerful technique for investigating the organization of the pairwise connections between nodes of networks. Application of graph theory to neuroimaging data reveals that in the normal human brain, regions tend to be connected in manner that creates an efficient ‘small world’ network in which long-range connections link or ‘integrate’ key local hubs that in turn connect to multiple nearby brain regions in a modular or segregated fashion. In patients with schizophrenia, the pattern of connections reveals a more segregated, less integrated and inefficient system (Bassett et al. 2008; Alexander-Bloch et al. 2010; Fornito et al. 2012; Zhang et al. 2012; van den Heuvel et al. 2013).

Among various morphological properties of the brain, cortical folding appears especially relevant to the development of brain as a connected system (Mota and Herculano-Houzel 2012; Chen et al. 2012). Experimental disruption of cortical connections during early stages of primate development produces alterations in folding patterns both proximal and distal to the induced lesions (Goldman-Rakic 1980; Goldman-Rakic and Rakic 1984). Thus the connections between regions can exert a strong influence on the cortical folding of  the connected regions, and it might be expected that the correlations between folding patterns in different brain regions would be informative of the development of cerebral connectivity (Neal et al. 2007; Takahashi et al. 2011). In light of the time locked patterns of fetal sulcation and gyrification (Dubois et al. 2008b; Nishikuni and Ribas 2013; Zhang et al. 2013), investigating disturbance of structural covariance patterns of cortical folding in adults could provide insights into neurodevelopmental aberrations.

To our knowledge, the structural covariance patterns of cortical gyrification are yet to be investigated in schizophrenia. In the present study, we applied graph theory to analyze the pattern of regional correlations in gyrification in patients with schizophrenia and in healthy controls to test the hypothesis that patients with schizophrenia would exhibit a greater degree of segregated architecture affecting key regional nodes such as the insula and the lateral prefrontal cortex, previously shown to have localizable cortical folding defects in patients (Palaniyappan and Liddle 2012). We also anticipated that patients with more severe illness would show a pronounced aberration in the connectomic architecture of cortical folding in these regions, implying a neurodevelopmental pathway to illness severity.

## Methods

### Subjects

The data reported in the present study were obtained from a previously reported (Palaniyappan and Liddle 2013) sample of 41 patients satisfying DSM-IV criteria for schizophrenia/schizoaffective disorder and 40 healthy controls. Patients were recruited from community-based mental health teams in Nottinghamshire and Leicestershire, United Kingdom. The diagnosis was made in a clinical consensus meeting in accordance with the procedure of Leckman et al. (1982), using all available information including a review of case files and a standardized clinical interview [Symptoms and Signs in Psychotic Illness—SSPI (Liddle et al. 2002)]. All patients were in a stable phase of illness with no change in antipsychotic, antidepressant, or mood-stabilizing medications in the 6 weeks prior to the study. Subjects with age <18 or >50, with neurological disorders, current substance dependence, or intelligence quotient <70 using Quick Test (Ammons and Ammons 1962) were excluded. The median defined daily dose (DDD) (WHO Collaborating Centre for Drug Statistics and Methodology 2003) was calculated for all prescribed psychotropic medications.

Healthy controls were recruited from the local community via advertisements, and 40 subjects free of any psychiatric or neurological disorder group matched for age and parental socioeconomic status [measured using National Statistics-Socio Economic Classification (Rose and Pevalin 2003)] included in the patient group. Controls had similar exclusion criteria to patients. In addition, subjects with personal or family history of psychotic illness were excluded. A clinical interview by a research psychiatrist was employed to ensure that the controls were free from current axis 1 disorder and history of either psychotic illness or neurological disorder. The study was given ethical approval by the National Research Ethics Committee, Derbyshire, United Kingdom. All volunteers gave written informed consent. Please see Table 1 for further sample characteristics.

**Table 1:** Demographic features of the sample

	Healthy controls (*n* = 40)	Patients with schizophrenia (*n* = 41)	*T*/*χ* ^2^
Gender (male/female)	29/11	31/10	*χ* ^2^ = 0.1, *p* = 0.8
Handedness (right/left)	36/4	37/4	*χ* ^2^ = 0.001, *p* = 0.97
Age in years (SD)	33.4 (9.1)	33.63 (9.2)	*T* = −0.12, *p* = 0.91
Mean parental NS-SEC (SD)	2.00 (1.3)	2.46 (1.5)	*T* = 1.46, *p* = 0.15
Global mean gyrification	2.99 (0.14)	2.95 (0.16)	*T* = 1.37, *p* = 0.18
Mean total SSPI score	–	11.7 (7.4)	
Reality distortion	–	2.24 (2.6)	
Disorganisation	–	1.34 (1.3)	
Psychomotor poverty	–	2.88 (3.8)	

### Assessment of clinical symptoms

For the patient group, we quantified current occupational and social dysfunction using the Social and Occupational Functioning Assessment Scale (SOFAS) (Goldman et al. 1992) and assessed speed of cognitive processing, a consistent and prominent cognitive deficit in schizophrenia using the Digit Symbol Substitution Test (DSST) (Dickinson et al. 2007). DSST was administered using a written and an oral format with a mean score computed from the two. In addition to current SSPI scores (on the day of MRI scan) to measure the symptoms of reality distortion, disorganization and psychomotor poverty, we also collected retrospective information regarding the longitudinal severity (persistence) of psychotic symptoms by applying the SSPI scale over using clinical case notes to derive a single numerical score representing total persistence of psychotic symptoms across the life course. High inter-rater reliability was achieved for the persistence measure among the three psychiatrists (VB, LP, RD) involved in this study (intra-class correlation coefficient = 0.87 (0.73−0.94); *n* = 25 subjects).

### Image acquisition

A magnetization-prepared rapid acquisition gradient echo image with 1 mm isotropic resolution, 256 × 256 × 160 matrix, repetition time (TR)/echo time (TE) 8.1/3.7 ms, shot interval 3 s, flip angle 8°, SENSE factor 2 was acquired for each participant using a 3T Philips Activa MR system.

### Gyrification analysis

Cortical surfaces were reconstructed using FreeSurfer version 5.1.0, employing standard preprocessing procedures as described by Dale et al. (1999). To measure cortical folding patterns for each of the several thousands of vertices across the entire cortical surface, we used the method advocated by Schaer et al. (2008) on the basis of an index originally proposed by Zilles et al. (1988). This method provides local gyrification indices (LGIs), numerical values assigned in a continuous fashion to each vertex of the reconstructed cortical sheet. The LGI of a vertex corresponds to the ratio of the surface area of the folded pial contour (“buried” surface) to the outer contour of the cortex (“visible” surface) included within spherical regions of interest (25 mm radius). This yielded a continuous gyrification surface map for each subject with each vertex on the reconstructed pial surface representing the LGI. This surface was then parcellated into 148 brain regions (74 in each hemisphere) using a sulcogyral atlas (Destrieux atlas) that follows the anatomical conventions of Duvernoy and allows separation of sulcal from gyral regions based on anatomical constraints of consistently occurring cortical folds (Destrieux et al. 2010). The average LGI of all vertices that were included in a parcellated region was assigned as the gyrification index value for the corresponding brain region.

### Constructing gyrification-based networks

**Fig. 1:**
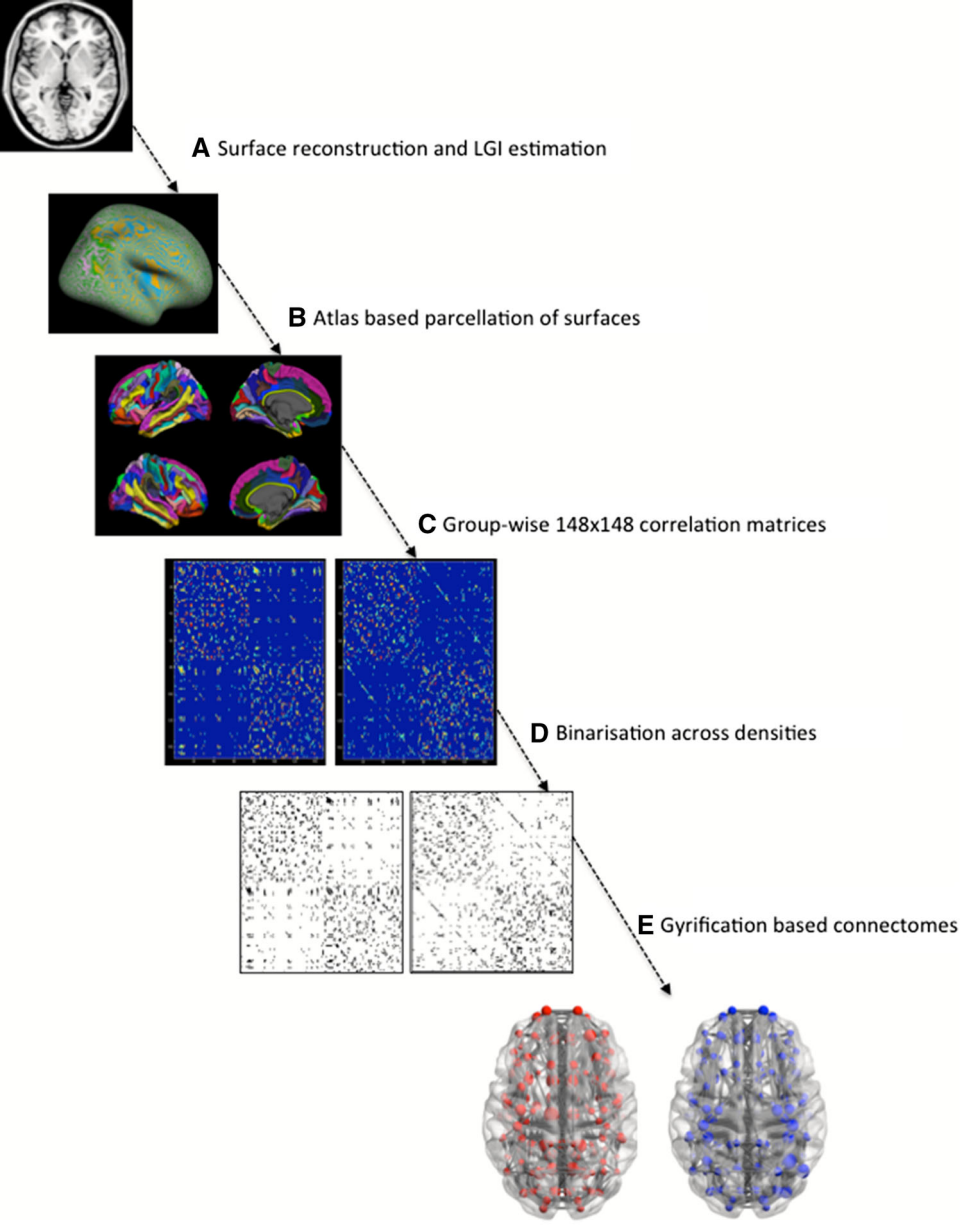
Steps in processing the gyrification-based networks. **a** Surface reconstruction was carried out using FreeSurfer software; local gyrification indices were estimated using Schaer’s procedure. **b** Destrieux atlas was used for parcellating the cortical surface to 148 regions (74 on each hemisphere). **c** Association matrices were obtained by calculating the correlations between regional gyrification across subjects within each group separately. **d** Binary adjacency matrices were derived from thresholding at minimum density for fully connected graphs in both groups

A 148 × 148 Pearson’s correlation matrix of gyrification indices of each parcellated brain region adjusted for age, gender, intracranial volume and mean overall gyrification index in line with He et al. (2007) was used to create a binary adjacency matrix for each group (CON and SCZ), using threshold values for the correlation coefficients. Instead of choosing a single coefficient threshold, we used a range of thresholds determined by connection densities (proportions of connections present in a graph to all possible connections) varying from 0.1 to 0.5 (increments of 0.05) to compare the properties of emerging networks. Across this range in both groups, the resulting graphs were fully connected and not fragmented (minimum density at which fully connected graph was observed = 0.08). The graphs approached random configuration beyond the density of 0.5. The steps involved in obtaining the networks are summarized in Fig. 1.

### Properties of the covariance networks

The patterns of relationship among brain regions within a network can be described using three groups of topological properties: segregation, integration and centrality (Stam and Reijneveld 2007; Bullmore and Sporns 2009; Rubinov and Sporns 2010). (1) *Integration* Shortest path length *L*_p_ between two regions (A, B) refers to the minimum number of connections that links A and B. If A and B have direct structural covariance, then they will have a direct connection in the gyrification network, with their *L*_p_ being 1. If A and B do not have direct covariance, but if A covaries with C, and C covaries with B, then the *L*_p_ between A and B will be 2 (mediated by 2 connections; AC and CB). The average shortest path length between all pairs of regions in the network gives the characteristic path length of the network (ML_p_). The inverse of ML_p_ is a measure of efficient information transfer, called as global efficiency *E*_glob_. (2) *Segregation* Clustering coefficient (*C*_p_) of a node is the number of existing links divided by the number of all possible links among the neighbors of a node. High *C*_p_ indicates a high degree of localized covariance. The average of clustering coefficients of each region (or node) provides the clustering coefficient of the network (MC_p_). Local efficiency of a region is a closely related metric given by the inverse of the shortest number of connections among each pair of neighboring regions. *C*_p_ and *E*_loc_ quantify the cliquishness of a region. (3) *Centrality* The degree centrality of a node is the number of connections between that node and all other nodes. This is a sensitive and readily interpretable measure of centrality for structural networks (Rubinov and Sporns 2010).

In a gyrification network, segregation or clustered covariance may suggest modular development or plasticity of related brain regions, indicating a potential for regionally selective functional dependency. On the other hand, integration or distributed covariance may result from maturational processes (or constraints) affecting the entire brain. A highly integrated gyrification network can also result from the presence of certain ‘central’ hub regions whose structure covaries with a large number of other brain regions, leading to widely distributed structural coupling. These three groups of topological properties (integration, segregation and centrality) can be quantified using various graph theoretical measures, as described above.

In line with previous connectomic studies, we estimated the small-world index by comparing the estimated topological properties (MC_p_ and ML_p_) of the two networks (CON and SCZ) with corresponding mean values of null random graphs (MC_null_ and ML_null_) constructed with same number of nodes, edges and degree distribution as the gyrification-based networks. Small-world index (SWI) is given by (MC_p_/MC_null_)/(ML_p_/ML_null_). SWI > 1 suggests a small-world network that has a relatively high segregation and integration compared to random null networks (Humphries and Gurney 2008). All topological properties were computed using Graph Analysis Toolbox (Hosseini et al. 2012) (http://brainlens.org/tools.html) that uses computation algorithms from Brain Connectivity Toolbox (https://sites.google.com/site/bctnet/). Further, we also used Newman’s optimization algorithm (Newman 2006) implemented in GAT to identify the modular organization in the CON and SCZ network. A module is a highly clustered community that can be defined as a subgroup of nodes with high propensity to form links within the subgroup rather than with regions outside the subgroup. For a given number of modules, the modularity value (*Q*) is defined as the difference between the numbers of intramodular links in a given network and the number of intermodular links that will be seen in a random network for same number of modules. Newman’s optimization algorithm detects the optimum number of modules that will give the highest possible *Q* for a given network. The networks were visualized using BrainNet Viewer (Xia et al. 2013) (http://www.nitrc.org/projects/bnv/).

### Group comparison

To test the statistical significance of the difference between the topological parameters of the two groups, non-parametric permutation test with 1,000 repetitions was employed. For each iteration, the entire set of regional gyrification indices (148 nodes) of each participant was randomly reassigned to one of two new groups with the sample size identical as CON and SCZ. This permutation approach preserves the gyrification index within regions but shuffles across individuals during resampling. Binary adjacency matrices across a range of network densities (0.1–0.5, increments of 0.05) were obtained for each randomized group. Topological measures were then calculated for the networks and differences between the random groups were computed across the entire range of densities. For the various topological properties, differences in the area under the curves obtained from plotting the values of each random group across the range of densities were obtained for each iteration. This resulted in a null distribution of differences, against which the p values of the actual differences in the curve functions obtained by comparing CON and SCZ were computed. This nonparametric permutation test based on functional data analysis (FDA) (Ramsay and Dalzell 1991) inherently accounts for multiple comparisons across the range of densities (Bassett et al. 2012; Singh et al. 2013). For regional (*n* = 148 nodes) properties such as local efficiency, clustering and degree, an additional correction for multiple comparison (false discovery rate) was used with corrected *p* < 0.01 considered as significance threshold. Hubs were defined as the nodes whose FDA-based curve function for regional degree is 2 standard deviations greater than the mean of corresponding curve functions obtained from the 1,000 random permutations.

### Relationship with illness severity

We performed a principal component analysis to extract the first unrotated principal factor explaining the largest proportion of variance from the measures of illness severity (3 SSPI syndrome scores, total persistence score, SOFAS score, DSST score). Positive loading of illness severity factor was seen in patients with persistent illness, poor functional ability, poor processing speed and higher symptom burden of disorganisation, psychomotor poverty and reality distortion. Negative loading indicated less persistent illness, with better functional ability, higher processing speed and lower symptom burden across the three syndromes. Based on the factor scores we divided the patient group into those showing greater illness severity (positive loading on the severity factor; *n* = 20) and less illness severity (negative loading on the severity factor; *n* = 21). Demographic features of these two groups are presented in the Supplementary Material. Gyrification networks were constructed and regional topological properties were compared for these two groups using the same approach employed for comparing healthy controls and patients.

## Results

**Fig. 2:**
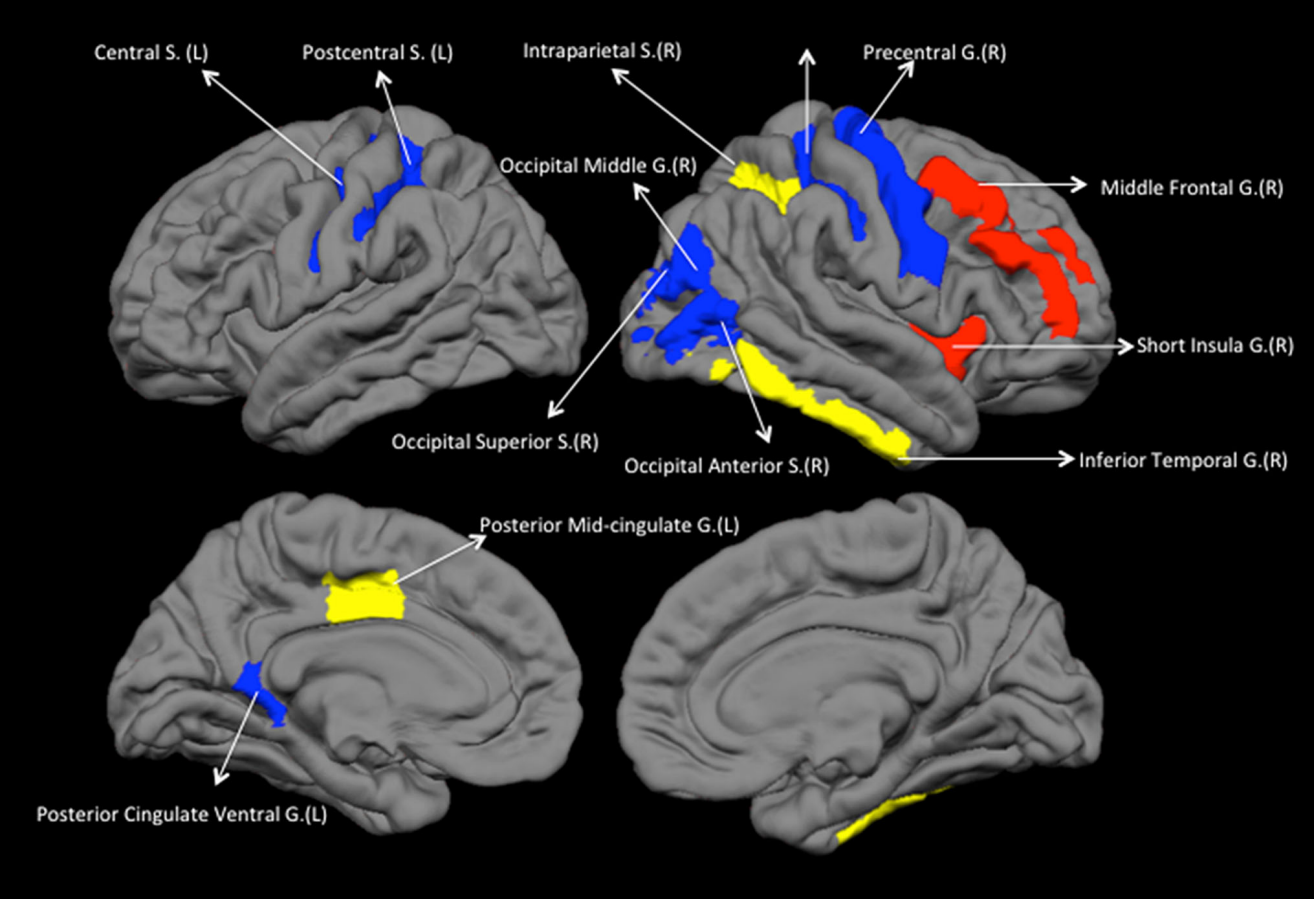
Regional changes in topological properties of the gyrification network. A *colour figure* is provided online. Middle frontal and short insula show increased segregation in patients with schizophrenia (increased in local efficiency/clustering coefficient). Inferior temporal, intraparietal and posterior midcingulate show decreased degree centrality in schizophrenia. All other labelled regions show reduced segregation in patients with schizophrenia. *L* left hemisphere, *R* right hemisphere, *G* gyrus, *S* sulcus

Both CON and SCZ networks showed small-worldness (mean SWI across densities for CON = 1.82; SCZ = 1.83). The overall segregation and integration measures of the two networks were not significantly different (Table 2) but comparison of individual nodal properties (Table 3) revealed significantly increased clustering coefficient for right anterior insula and reduced clustering coefficient for several regions in the right occipital cortex and bilateral central sulcus in SCZ compared to CON. Left posterior cingulate gyrus also showed reduced clustering in SCZ. Local efficiency was significantly increased for right middle frontal gyrus, and reduced in bilateral central and postcentral sulcus for SCZ compared to CON. These results are summarized in Fig. 2. In CON, all of the 5 hub regions were located in the anterior cingulate cortex; while in SCZ no nodes had degree centrality that satisfied the criteria for hubs (>2 SD of the mean).

**Table 2:** Topological properties of gyrification-based connectome

	Controls	Schizophrenia	FDA permutation test (*p* values)
Measures of segregation			
Clustering coefficient cp	0.5734	0.5459	0.45
Mean local efficiency	0.7774	0.7643	0.46
Measures of integration			
Characteristic path length	1.886	1.863	0.63
Global efficiency	0.619	0.622	0.68
Hubs based on degree centrality			
Left anterior cingulate		NA	NA
Left anterior midcingulate			
Left posterior midcingulate			
Right anterior cingulate			
Right anterior midcingulate

**Table 3:** Regional topological properties altered in schizophrenia

Nodes with altered local clustering coefficient		
SCZ > CON	Right insula short gyrus	0.006
CON > SCZ	Right postcentral sulcus	0.002
	Right central sulcus	0.002
	Right occipital anterior sulcus	0.004
	Left posterior cingulate gyrus (ventral)	0.004
	Right occipital superior/transversal sulcus	0.006
	Right precentral gyrus	0.008
	Right occipital middle gyrus	0.01
Nodes with altered local efficiency		
SCZ > CON	Right frontal middle gyrus	0.002
CON > SCZ	Right central sulcus	0.005
	Left central sulcus	0.006
	Right postcentral gyrus	0.002
	Left postcentral sulcus	0.01
Nodes with altered degree		
SCZ > CON	None	NA
CON > SCZ	Right inferior temporal gyrus	0.007
	Right intraparietal and transverse parietal	0.002
	Left posterior midcingulate	0.001

*SCZ* patients with schizophrenia *CON* healthy controls

**Fig. 3:**
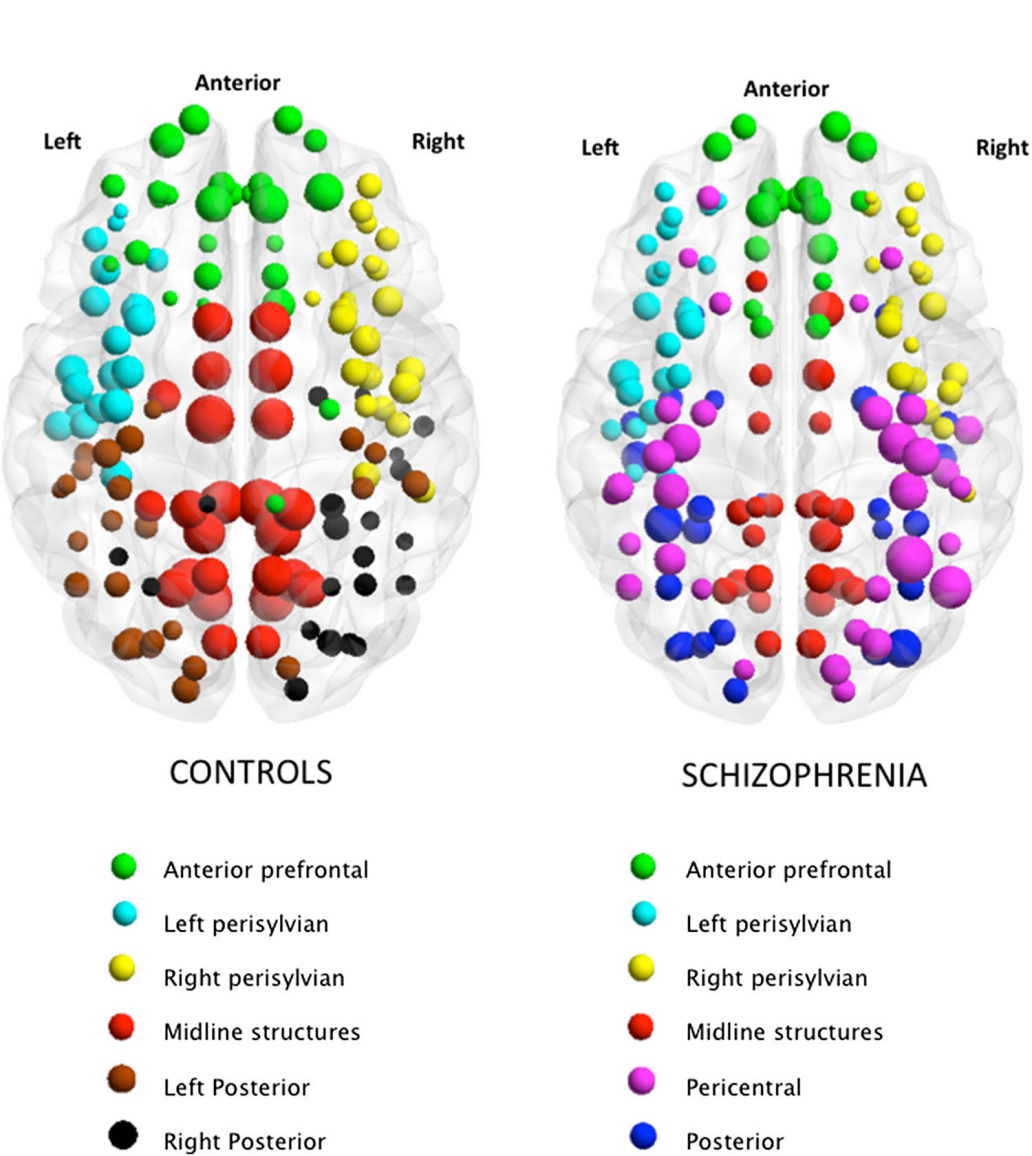
Graphical representation of gyrification networks in controls (CON) and patients with schizophrenia (SCZ), visualized using BrainNet viewer (http://www.nitrc.org/projects/bnv). Both CON and SCZ networks had 6 modules each discovered using Newman’s module detection algorithm, coded separately for each network. The size of the nodes is proportional to the degree centrality. A *colour figure* showing module membership of individual nodes is provided online

In both CON and SCZ groups, 6 optimized modules were noted. The distribution of the module membership in controls revealed two perisylvian and two posterior (lateral parieto-temporo-occipital) modules on either hemisphere along with a medial module for midline structures and an anterior prefrontal module. In patients, the two perisylvian, the medial (midline structures), and the anterior prefrontal modules were mostly preserved. A combined pericentral module was noted in patients, which included some lateral frontal and lateral parietal nodes that were clustered with either prefrontal or the posterior module in controls. A single posterior module was seen in patients that included several structures from the right and left posterior modules in the controls. The modular structure of the network is shown in Fig. 3. The degree distribution of the two networks is presented in Supplementary Material.

Patients with greater severity of illness had significantly increased clustering coefficient and local efficiency in several nodes including the right insula, superior temporal and inferior frontal cortex. Further results from this analysis are presented in Table 4.

**Table 4:** Regional topological properties in association with illness severity

Nodes with altered local clustering coefficient		
High > Low	Right circular insula sulcus inferior	0.005
	Right insula short gyrus	0.01
	Right superior temporal gyrus	0.01
	Right inferior frontal (pars triangularis) gyrus	0.01
Low > High	Left angular gyrus	0.01
	Right occipital anterior sulcus	0.01
Nodes with altered local efficiency		
High > Low	Right circular insula sulcus inferior	0.01
	Right circular insula sulcus superior	0.01
	Right short insula gyrus	0.01
	Right superior temporal gyrus	0.01
Low > High	NA	
Nodes with altered degree		
High > Low	Left frontal inferior orbital gyrus	0.01
	Left lateral fusiform gyrus	0.01
Low > High	Right inferior frontal sulcus	0.002

High: 20 subjects with positive loadings. Low: 21 subjects with negative loadings on illness severity factor. The severity factor was derived from the scores on 3 SSPI syndromes (reality distortion, psychomotor poverty and disorganization), total persistence, SOFAS and DSST scores

## Discussion

To our knowledge, we report the presence of robust small-world properties in the gyrification-based network for the first time in both healthy controls and in schizophrenia. Presence of small-worldness in gyrification-based network suggests that even in the absence of a direct covarying relationship in folding patterns between some brain regions, a small number of other regions mediate the overall interrelatedness, with the folding pattern of the entire brain showing a complex relationship typical of several evolutionarily advantageous biological networks (Bullmore and Sporns 2012). Importantly, this also suggests that while most of the structural covariance in cortical folding is limited to proximal nodes (clustering), gyrification patterns of distal regions are also strongly interrelated. Despite the absence of prominent alterations in the global efficiency, we note significant alterations in the regional topological properties, with increased segregation of right anterior insula and right middle frontal (dorsolateral prefrontal) gyrus along with a reduction in the segregation of structures around the central sulcus and lateral occipital cortex. In addition, the prominent centrality of cingulate structures that was observed in healthy controls was not present in patients.

The overall small-world architecture of the gyrification network is preserved in schizophrenia, suggesting that abnormalities in the folding patterns seen in patients are subtle and do not affect the basic organizing principles of cortical folding. Nevertheless, patients with schizophrenia showed significant changes in the regional topological properties. Right anterior insula and right dorsolateral prefrontal region were highly segregated with more localized covariance in patients than controls. These two regions belong to two distinguishable large-scale networks that form an integrated information processing system (Seeley et al. 2007; Menon and Uddin 2010). Several neuroimaging studies have repeatedly implicated the importance of these two regions in a myriad of cognitive processing tasks, highlighting the relative importance of these two regions in enabling efficient coordination with the rest of the brain (Critchley et al. 2004; Bressler and Menon 2010). Abnormalities in the functional connectivity pertaining to these regions have been repeatedly observed in patients with schizophrenia (Moran et al. 2013; Manoliu et al. 2013; Palaniyappan et al. 2013b, c). Reduced gyrification of insula and dorsolateral frontal cortex has also been previously reported in schizophrenia (Bonnici et al. 2007; Nesvåg et al. 2014). Abnormally increased segregation of these regions suggest that in patients, the developmental trajectory of the right anterior insula and the middle frontal gyrus has relatively less influence on distributed brain regions, but more influence on anatomically constrained proximal regions. Altered localized covariance or cliquishness could be due to an aberrant developmental process that affects all of the neighboring brain regions that are highly connected to these structures. Alternatively, processes affecting plasticity such as learning or training in association with repeated and excessive recruitment can bring about an increased covariance within the clustered regions, though such effects have not been directly demonstrated so far. In this context, the increased segregation of these regions can be also  interpreted as a compensatory process (Griffa et al. 2013).

Patients had a reduction in segregation and local efficiency in primary sensory regions such as the structures around the central sulcus and occipital cortex. These findings are somewhat unexpected given that whole brain univariate approaches have hitherto not identified prominent folding deficits in these regions. Structural covariance studies in adolescents report a developmental reduction in the local efficiency of primary sensory regions (Alexander-Bloch et al. 2013a) In general, primary sensory (and motor) regions show much more segregated developmental pattern than association cortices in healthy controls (Raznahan et al. 2011; Li et al. 2013). Notably, patients with early onset schizophrenia show accelerated grey matter loss around the central sulcus (Gogtay et al. 2004). These changes have been ascribed to disturbances in synaptic pruning in schizophrenia, though no direct evidence exists to date to confirm or refute this notion. In-so-far as the tension related to neuronal connections determines cortical folding (Essen 1997; Hilgetag and Barbas 2006), excessive pruning of such connections, if it indeed occurs in schizophrenia, can also alter the anatomical covariance patterns in gyrification.

In the present sample, healthy controls had a high-degree centrality involving several cingulate regions. This suggests that the gyrification of the cingulate cortex correlates with a large number of other brain regions in healthy state, but not in schizophrenia. Consistent with these observations, alterations in cingulate morphology have been reported previously in schizophrenia (Wheeler and Harper 2007; Baiano et al. 2007). Furthermore, the visual inspection of the modularity and degree distributions patterns (Fig. 3) reveals that various midline structures show a reduction in their degree of covariance in patients. In addition, the inferior temporal and superior parietal sulcus also show reduced degree, while there were no regions showing increased degree in patients. This implies that the pathophysiological process that characterizes gyrification defects in schizophrenia predominantly reduces the overall structural covariance patterns.

Patients with greater illness severity display a more segregated pattern of covariance especially for the right anterior insula extending to include the right superior temporal gyrus and inferior frontal gyrus (pars triangularis). These structures are right homologues of critical language-processing regions that are repeatedly implicated in the generation of psychotic symptoms (Jardri et al. 2011; Li et al. 2012; Modinos et al. 2013). This is consistent with a recent observation indicating that abnormal fronto-temporo-insular gyrification may predict poor outcome in psychosis (Palaniyappan et al. 2013b). Taken together, these results support a speculation that in the presence of a well-coordinated development of the peri-sylvian regions, especially the anterior insula, the longitudinal course of schizophrenia could turn out to be more favorable. Understanding the factors that influence the maturation of these brain regions in health and disease states could provide opportunities to modify illness trajectories in future. To our knowledge, this is the first time that the topological properties of the structural graph networks from patients with schizophrenia are shown to be related to severity of clinical symptoms (van den Heuvel et al. 2010; Fornito et al. 2012), highlighting the utility of studying gyrification patterns in this illness.

Our study has a number of strengths. We adopted a whole brain approach to study structural covariance, instead of seed-based or subset-based approaches. This data-driven approach obviates the need for generating region-based hypotheses that are likely to be tenuous given the heterogeneity of results from previous studies in schizophrenia (White and Gottesman 2012). We defined nodes based on parcellations derived from a sulcogyral atlas that is based on anatomical boundaries of consistent sulci and gyri (Destrieux et al. 2010). There is no consensus on the choice of nodes for connectomic studies; the absolute values of small-world properties have been reported to vary significantly according to the size of the nodes (Zalesky et al. 2010; Fornito et al. 2013). Nevertheless, the use of a common spatial scale for group comparison has been shown to provide valid results (Evans 2013). In line with other anatomical covariance studies, we used population-level variability to determine topological properties for each group; as a result, topological measures at an individual level were not available to relate to symptom burden or cognitive scores. Nevertheless, we have used a median-split approach to study subgroups with varying clinical severity to establish the relationship with symptom burden. We studied a sample of medicated patients; antipsychotic use is reported to be associated with structural changes in schizophrenia (Ho et al. 2011), though at present there is no evidence that suggests that cortical folding patterns are affected by the use of antipsychotics. Our investigation of the linear association between antipsychotic dose and topological properties (Supplementary Material) suggested that though antipsychotics have some influence on the covariance pattern, this is not sufficient to affect the topological properties of the gyrification-based network. However, our findings must be interpreted cautiously until replicated in a sample of untreated patients.

Abnormalities in the covariance patterns involving the frontal cortex are of particular interest for the study of schizophrenia. Phylogenetic variations in sulcal patterns predominantly involve the frontal cortex, suggesting a link between cognitive/linguistic evolution and cortical folding (Zilles et al. 2013). To our knowledge, there are no comparative studies on the structural covariance of gyrification across species. Within-species variations in gyrification appear to be influenced by factors that exert region-specific effects on the brain (Kochunov et al. 2010). These factors influence the ontogeny of cerebral gyrification and may relate to the structural covariance. Broadly, we can group the factors influencing gyrification as fetal events, early infantile events and later developmental events (including the maturational changes related to puberty). Distribution of regional axonal tension (in fetal, infantile or later life periods), differential rates of surface expansion (especially in fetal and infantile period) and genetic variations in cellular proliferation and migration (occurring in fetal life) could influence the establishment of specific patterns of structural covariance in cortical folding (Zilles et al. 2013).

Interestingly, a substantial portion of within-species variance in gyrification patterns appears to be non-genetic (Rogers et al. 2010; Zilles et al. 2013), highlighting the role of non-heritable, possibly late maturational events. Synchronized recruitment of brain regions can *induce* structural covariance through use-dependent synaptogenesis even in the absence of direct axonal connectivity (Evans 2013). Mindfulness meditators, who repeatedly use the interoceptive brain regions such as the insula and the salience network structures, show increased insular folding in direct relationship to the duration of their meditative practice (Luders et al. 2012). Our cross-sectional design precludes further parsing of the factors operating at different stages of life to influence the structural covariance. Nevertheless, it is important to note that the largest transformation in cortical folding patterns in one’s lifespan occurs during fetal life (White et al. 2010). During this time, a well-coordinated spatial relationship in sulcogyral development is seen across the brain (Habas et al. 2012). This suggests that a substantial amount of the structural covariance observed in later life is determined during this period. Events that disrupt fetal neurodevelopment significantly affect cortical folding (Dubois et al. 2008a; b), and its relationship with white matter connectivity (Lodygensky et al. 2010; Melbourne et al. 2014), affecting later functional capacity in adult life (Kesler et al. 2006; Dubois et al. 2008a). This supports our interpretation that the prominent reshuffling of the gyrification covariance (Fig. 2) is an early developmental aberration in brain connectivity in patients. To conclusively differentiate the early developmental influences from the later life events, a prospective study of prenatal or newborn cohorts followed up till late adult life, ideally even after the onset of schizophrenia, is required.

The use of graph theoretical approach has revealed novel insights about the complex connected system of relationship among different brain regions in health and its aberration in disease states (Johansen-Berg 2013). Several years of neuroimaging research in schizophrenia with ‘lesional’ approaches has uncovered some, but most of the pathophysiological processes associated with the clinical picture of schizophrenia remains yet to be discovered (Carpenter et al. 1993; Tandon et al. 2008). This approach, when used to study cortical folding patterns for the first time, reveals significantly altered covariance, suggesting abnormalities in the developmental synchrony of connected brain regions in patients. In particular, our study has identified specific regions, whose coordinated maturation with the rest of the brain may be specifically altered in schizophrenia, contributing to greater severity. Longitudinal and interventional (e.g. motivated cognitive training) studies of cortical gyrification that specifically focuses on these brain regions are likely to uncover a more complete picture of the structural substrate of dysconnectivity that characterizes schizophrenia. Given the emerging evidence implicating the importance of cortical folding defects in treatment response future studies utilizing gyrification-based covariance approaches could aid in further characterizing poor-outcome phenotype in psychotic disorders.

## Electronic supplementary material

Below is the link to the electronic supplementary material.

Supplementary material 1 (DOCX 260 kb)
