# Supplementary material for: Abnormalities in structural covariance of cortical gyrification in schizophrenia
Source: Brain Struct Funct. 2014 Apr 26;220(4):2059–71. doi: 10.1007/s00429-014-0772-2 (PMC4481329; doi:10.1007/s00429-014-0772-2)
Supplement: Supplementary file 1 — Supplementary material 1 (DOCX 260 kb) [file 429_2014_772_MOESM1_ESM.docx]

**Supplementary Material**

**CONTENTS**

1. Degree Distribution Analysis (Pg 2)
2. Demographic features of the subgroups based on the severity of illness Table 1S (Pg 3)
3. Effect of antipsychotics on topological properties - Table 2S, Table 3S, Figure 1S and Figure 2S (Pg 4)

**Degree distribution analysis**

The degree distributions of brain connectivity networks often follow an exponentially truncated power-law function that can be expressed as

P(d) ~ [d^(e-1)^ * exp^(-d/dc)^]

P(d) is the probability of regional degree (d)

dc is the cut-off degree above which there is an exponential decay in probability of hubs

e is the exponent.

If such a truncated power-law relationship can be demonstrated for a connectome, this will indicate the presence of a scaling regimen, followed by an exponential decay in the probability of finding nodes whose degree is greater than the cutoff value.

For both controls and patients, we indeed observed an exponentially truncated power-law distribution. The exponent estimate (e) was 1.70 for SCZ and 1.80 for CON networks. The cut-off degree (dc) was 6.43 for SCZ network and 5.79 for the CON. The R-square value for the distribution fit was 0.88 for SCZ and 0.90 for CON, suggesting the truncated power-law model had a very good fit for the data.

These observations suggest that the gyrification networks in both groups included some hub nodes with high degree of coupling with several other brain regions, but are constrained from the emergence of very high degree hubs that could affect the resilience of the system. This further validates that the gyrification-based network has topological properties reminiscent of other connectivity-based graph networks (connectomes) obtained from neuroimaging data.

**Table 1S: Demographic features of the subgroups based on the severity of illness**

|  | **Less severity (n=21)** | **High severity (n=20)** | **T/X2** |
| --- | --- | --- | --- |
| **Gender (male/female)** | 13/8 | 18/2 | x2=2.99, p=0.08 |
| **Handedness (right/left)** | 19/2 | 18/2 | x2=0.00, p=1.0 |
| **Age in years (SD)** | 31.4(9.1) | 35.9 (9.1) | T=-1.6 p=0.12 |
| **Parental NS-SEC (SD)** | 1.9(1.3) | 3.1(1.5) | T=-2.8, p=0.01 |
| **Global mean gyrification** | 2.97(0.17) | 2.93(0.15) | T=0.79, p=0.43 |
| **DDD (SD)** | 1.2(1.03) | 1.4(1.2) | T=-0.58, p=0.56 |
| **Variables included in PCA to derive illness severity factor** | | | |
| **SOFAS (SD)** | 62.0(11.39) | 46.9(10.05) |  |
| **DSST (SD)** | 47.6(8.12) | 37.1(8.39) |  |
| **Total persistence (SD)** | 10.7(3.16) | 15.1(6.3) |  |
| **SSPI Reality Distortion** | 1.3(1.96) | 3.2(2.8) |  |
| **SSPI Disorganisation** | 0.6(0.8) | 2.2(1.22) |  |
| **SSPI Psychomotor Poverty** | 0.7(1.14) | 5.2 (4.3) |  |

PCA: Principal Component Analysis SSPI: Signs and Symptoms in Psychotic Illness SOFAS: Social Occupational and Functional Assessment Scale DSST: Modified Digit Symbol Substitution Test DDD: Define Daily Dose of antipsychotics. NS-SEC: Parental Socio-Economic Status (National Statistics Scale) SD: Standard Deviation

**Effect of antipsychotics on topological properties**

**Figure 1S: Current antipsychotic dose effect:** Correlation matrices for patient sample thresholded at minimum density for full connectivity. A) Matrix without adjusting for the effect of current antipsychotic dose. B) Matrix linearly adjusted for the effect of current antipsychotic dose. Color bar indicates absolute correlation coefficients (varying from 0 to 1). C) Absolute matrix obtained from subtracting A and B.

While most pairwise correlations appear unaffected by antipsychotic dose, there is some linear relationship between the prescribed dose and structural covariance. To further investigate this effect, we obtained the global network topological measures at the minimum density of full connectivity for both antipsychotic-adjusted and non-adjusted networks. Comparison of these values using the same permutation approach described in the manuscript did not reveal any significant differences between the two networks (all p>0.6; Table 2S).

**Table 2S: Effect of current dose of antipsychotics on topological properties**

|  | **Network unadjusted for daily antipsychotic dose** | **Network linearly adjusted for daily antipsychotic dose** |
| --- | --- | --- |
| **Measures of Segregation** | | |
| Clustering Coefficient | 0.526 | 0.518 |
| Mean Local Efficiency | 0.724 | 0.716 |
| **Measures of Integration** | | |
| Characteristic Path Length | 2.710 | 2.705 |
| Global Efficiency | 0.430 | 0.430 |

Cumulative exposure to antipsychotics is likely to be more influential on the brain morphology than the current stable dose. In the current study, we did not have the longitudinal information on cumulative dose prescription or intake. Further, we also lacked any data on the individual concordance levels of the prescribed antipsychotics. Therefore, we sought to study the relationship between structural covariance of gyrification and cumulative antipsychotic exposure, using a product of define daily dose (DDD) and duration of illness since the time of first presentation with psychotic episode, determined from patients’ case notes. This index can be taken as an approximate measure of lifetime antipsychotic exposure (ALAE). We repeated the above analysis by linearly adjusting for ALAE when constructing the covariance networks.

**Figure 2S:** **Approximate lifetime antipsychotic exposure effect:** Correlation matrices for patient sample thresholded at minimum density for full connectivity. A) Matrix without adjusting for the effect of approximate lifetime antipsychotic exposure. B) Matrix linearly adjusted for approximate lifetime antipsychotic exposure. Color bar indicates absolute correlation coefficients (varying from 0 to 1). C) Absolute matrix obtained from subtracting A and B.

We observed that most pairwise correlations were unaffected by ALAE, with very few relationships being influenced by the cumulative exposure to antipsychotics. To further investigate this effect, we obtained the global network topological measures at the minimum density of full connectivity for both ALAE-adjusted and non-adjusted networks. Comparison of these values using the same permutation approach described in the manuscript did not reveal any significant differences between the two networks (all p>0.53; Table 3S). Note that the absolute values in Table 2S and 3S will differ due to the variation in minimum density levels for full connectivity for each comparison.

**Table 3S: Effect of approximate lifetime exposure of antipsychotics on topological properties**

|  | **Network unadjusted for daily antipsychotic dose** | **Network linearly adjusted for daily antipsychotic dose** |
| --- | --- | --- |
| **Measures of Segregation** | | |
| Clustering Coefficient | 0.492 | 0.483 |
| Mean Local Efficiency | 0.701 | 0.696 |
| **Measures of Integration** | | |
| Characteristic Path Length | 2.642 | 2.581 |
| Global Efficiency | 0.437 | 0.442 |

This suggests that the few brain regions whose structural covariance in gyrification relate to antipsychotic use are not sufficient to affect the topological properties of the gyrification-based covariance networks. Of note, there has been only one study where longitudinally recorded cumulative antipsychotic exposure was related to gyrification indices in schizophrenia (Palaniyappan et al. 2013). This study found no appreciable association between antipsychotic exposure and gyrification patterns. This supports our inference that the covariance in gyrification noted in the current study is likely to be related to the pathophysiology of schizophrenia rather than being an effect of the antipsychotic treatment.

**Reference**

Palaniyappan L, Crow TJ, Hough M, et al. (2013) Gyrification of Broca’s region is anomalously lateralized at onset of schizophrenia in adolescence and regresses at 2 year follow-up. Schizophr Res 147:39–45. doi: 10.1016/j.schres.2013.03.028
